# MicroRNA-9-5p regulates apoptosis of human osteoarthritis chondrocytes through suppressing TnC

**DOI:** 10.4314/ahs.v25i1.16

**Published:** 2025-03

**Authors:** Xinming Yi, Jun Lei, Ye Hua, Chao Chen, Jun Yang

**Affiliations:** 1 Department of Orthopedics, Affiliated Hospital of Xinyang Vocational and Technical College, Xinyang, China; 2 College of Pharmacy, Xinyang Vocational and Technical College, Xinyang, China

**Keywords:** microRNA-9-5p, apoptosis, osteoarthritis, TnC

## Abstract

**Background:**

Osteoarthritis (OA) is one of the most common degenerative diseases, with obesity being one of the main contributing factors. MicroRNAs (miRNAs) have been shown to regulate gene expression and improve OA, making them a promising target for future treatment strategies.

**Methodology:**

We measured the expression levels of miR-9-5p and Tenascin C (TnC) using quantitative Real-time PCR. We examined the effect of miR-9-5p overexpression/silencing on chondrocytes using Western blot analysis to assess protein levels and quantitative Real-time PCR analysis to assess gene expression of TnC, Bax, and Bcl-2. We also performed a dual luciferase reporter gene assay to investigate the correlation between miR-9-5p and TnC targeting regulation.

**Results:**

We validated successful overexpression/inhibition of miR-9-5p compared to negative control (NC) using quantitative Real-time PCR. We found that the expression of TnC and Bax protein was significantly decreased in the mimic+Model group compared to the model group, while Bcl-2 protein was significantly increased in the mimic+Model group. In contrast, we observed the opposite effect in the inhibitor+Model group. Moreover, our results suggested that upregulation of miR-9-5p decreased the expression of inflammatory factors.

**Conclusion:**

miR-9-5p inhibits chondrocyte apoptosis by targeting TnC, offering a therapy target for OA treatment. Our study indicates that miR-9-5p plays an important role in the regulation of OA chondrocytes and can inhibit cell apoptosis by negatively targeting TnC, providing a promising therapy target for the treatment of OA.

## Introduction

Osteoarthritis (OA), a common degenerative disease affecting more than 655 million people worldwide, is characterized by articular cartilage fibrosis and degeneration, osteophyte formation, subchondral osteosclerosis, and synovial tissue hyperplasia, which can significantly impair a patient's self-care and labor abilityOsteoarthritis (OA) is one of the most common degenerative diseases, affecting more than 655 million people worldwide^1^. The main manifestations of OA are articular cartilage fibrosis and degeneration, osteophyte formation, subchondral osteosclerosis and synovial tissue hyperplasia, seriously damaged the patient's self-care and labor ability^2^,^31^-^3^. Degradation of extracellular matrix (ECM), increased expression of inflammatory cytokines, and apoptosis mediators have been suggested to contribute to OADegradation of extracellular matrix (ECM), increased expression of inflammatory cytokines and apoptosis mediators may contribute to OA^4^,^5^. The current treatment options for OA, such as weight loss, oral and local use of NSAIDs, and intra-articular treatment, have limited effectiveness and durationThe treatment methods for OA mainly include weight loss, oral and local use of NSAIDs, intra-articular treatment, etc., but it is difficult to obtain effective and lasting effects^6^. Recent research has shown that the development of OA may be related to epigenetic mechanisms. Recent research shows that the development of OA is related to epigenetic^7^. Epigenetics is mainly divided into three categories: DNA modification, histone modification, and non-coding RNA species, non-coding RNA species generally refers to DNA methylation, histone acetylation, and miRNA regulation, abnormal expression of miRNA will lead to changes in gene expression^8^-^10^. Therefore, it is still urgent to elucidate the potential regulatory mechanism of abnormal gene expression in OA.

MicroRNA are endogenous non-protein coding RNAs that are 19-25 bases long and highly conserved, microRNA regulated gene expression through targeteing mRNAs^11^. Which affects cell proliferation, differentiation, apoptosis and apoptosis ontogeny, etc.^12^. More and more studies have shown that miRNA plays an important role in the pathological development of OA^13^,^14^. Although more and more studies have proved that miR-9-5p plays an important role in cancer progression, There is also a correlation between miR-9-5p and OA^15^, However, there is still a lack of research on miR-9-5p and OA, the possible role and potential mechanism of miR-9-5p in OA are still unclear.

In our research, we aimed to study that miR-9-5p regulates the development of OA. We first treated chondrocytes with the inflammatory mediator IL-1β to establish an in vitro OA model and confirmed that miR-9-5p expression was downregulated, however, the expression of TnC was upregulated. TnC could inhibit cell apoptosis and inflammation. In conclusion, our findings substantiated the role of miR-9-5p in OA and helped clarify the potential use of biomarkers and potential therapeutic targets.

## Materials and methods

### Cell Culture

Primary human chondrocytes were purchased from Pricella (Wuhan, ChinaHuman hondrocytes and HEK 293T cells were purchased from Pricella (Wuhan, China)), HEK 293T cells were cultured in Dulbecco modified Eagle medium (DMEM; KeyGEN BioTECH, Jiangsu, China) containing 10% fetal bovine serum (FBS; Gibco, Carlsbad, CA, USA) 100 mg/mL streptomycin and 100 IU/mL penicillin and. Primary human hondrocytes and chondrocyte culture medium were purchased from Pricella (Wuhan, China), which human hondrocytes were cultured in chondrocyte culture medium. Use recombinant human interleukin IL-1β (Novoprotein Technology, Jiangsu, China) stimulated human chondrocytes at a concentration of 10 ng/ml for 24 hours to establish an in vitro model of OA.

### Cell transfection

MiR-9-5p mimics, miR-9-5p inhibitors, miR-9-5p mimic NC and miR-9-5p inhibitor NC were synthesized and obtained from ZHBY Biotech (Jiangxi, China). According to the protocol of the kit, chondrocytes were transfected with 50 nM of miR-9-5p mimics or inhibitors using Lipofectamine 3000 Transfection Reagent for 48 hourschondrocytes were transfected with Lipofectamine 3000 Transfection Reagent (Invitrogen, Carlsbad, CA, USA).

### Cell counting kit-8 (CCK-8)

Cell viability was examined by cell counting kit-8 assay (CCK-8, Beyotime, Shanghai, China) Specifically, transfected chondrocytes were transferred into a 96-well plate (8,000 cells/well) and treatment with 10 ng/ml IL-1β 24 hours, 10 µL/well CCK-8 reagent was added into chondrocytes and cultured for 2 h at 37°C. Subsequently, the OD value of the cells at 450 nm was measured with an automatic microplate reader (WD-2102B, LIUYI BIOTECHNOLOGY, Beijing, China).

### Enzyme-Linked Immunosorbent Assay

Collect the cell supernatant and subsequently according to the manufacturer's protocol, use the corresponding ELISA kit (MEIMIAN, Jiangsu, China) to determined IL-6 and TNF-α in supernatant of cell culture. Calculate the actual concentration of the cell supernatant according to the linear regression curve of the standard sample. Verify the cell modeling and the expression of inflammatory factors after transfection.

### Flow cytometry

Chondrocytes apoptosis rates was determined by Annexin V-FITC/PI Apoptosis Kit (MULTI SCIENCES, Hangzhou, China), and then, chondrocytes apoptosis was analyzed with a flow cytometer fluorescence-activated cell sorting (FACS) (NovoCyte 2060R, ACEA, Zhejiang, China).

### Quantitative real-time PCR (qRT-PCR)

The cells were harvested after 48 h transfection, and the total RNA was extracted using TRIzol Reagent (CWBIO, Jiangsu, China). According to the manufacturer's protocol, RNA was reversely transcribed into cDNA using miRNA 1st Strand cDNA Synthesis Kit (Vazyme, Nanjing, China), qRT-PCR was performed on CFX Connect™(Bio-Rad, Shanghai, China) with HiScript II Q RT SuperMix for qPCR (+gDNA wiper) (Vazyme, Nanjing, China). The real-time quantitative PCR reaction conditions were as follows: 95°C, 10 min, 95°C, 10 s, 58°C, 30 s, 72°C, 30 seconds. A total of 40 cycles were amplified. U6 was regarded as an internal reference for miR-9-5p, and β-actin was regarded as an internal reference for other genes. The 2-ΔΔCt method was used to calculate the relative expression of genes (Table 1).

**Table 1:** The qRT-PCR sequences

Gene Name	Sequences (5′-3′)
β-actin Forward	TGGCACCCAGCACAATGAA
β-actin Reverse	CTAAGTCATAGTCCGCCTAGAAGCA
U6 Forward	CTCGCTTCGGCAGCACA
U6 Reverse	AACGCTTCACGAATTTGCGT
miR-9-5p Forward	GCGCGTCTTTGGTTATCTAGCT
miR-9-5p Reverse	AGTGCAGGGTCCGAGGTATT
TnC Forward	AGACAGATAACAGCATCACCCT
TnC Reverse	GGAACATCAACCTCAGCGT
Bax Forward	GGATGCGTCCACCAAGAA
Bax Reverse	AAAGTAGAAAAGGGCGACAAC
Bcl-2 Forward	GAGGATTGTGGCCTTCTTTG
Bcl-2 Reverse	GCCGGTTCAGGTACTCAGTC

### Western Blot

Total protein samples were extracted by radioimmunoprecipitation assay (RIPA) buffer (ApplyGen, Beijing, China). A total of 30 µg protein were separated by 10% sodium dodecyl sulphate-polyacrylamide gel electrophoresis (SDS-PAGE) and transferred to polyvinylidene fluoride (PVDF) membranes (Millipore, Billerica, MA, USA). Those membranes were incubated using 5% non-fat milk in tris buffered saline-tween (TBST) buffer and incubated with primary antibodies including Mouse Anti-β-Actin (TransGen Biotech, Beijing, China, 1/2000), Mouse Anti TnC (Proteintech, Rosemont, IL, USA, 1/1000), Rabbit Anti Bax (Proteintech, Rosemont, IL, USA, 1/1000), Rabbit Anti Bcl-2 (Bioss, Woburn, MA, USA, 1/1000). Besides, these membranes were incubated using secondary antibody (HRP conjugated Goat Anti-Mouse IgG (H+L) (Servicebio, Wuhan, China, 1/2000) and HRP conjugated Goat Anti-Rabbit IgG (H+L) (Servicebio, Wuhan, China, 1/2000)). And then, visualized the protein in PVDF membranes were using an ECL detection system (Tanon-5200).

### Luciferase Reporter Assay

The 3′-UTR luciferase reporter construct of TnC to form WT-TnC or mut-TnC, respectively. Then, miR-9-3p mimic or miR-NC mimic were transfected into 293T cells with Lipofectamine 3000 Transfection Reagent (Invitrogen, Carlsbad, CA, USA) in accordance with the manufacturer's protocol. Furthermore, according to the manufacturer's protocol, use the Promega Corporation (Madison, WI, USA) to examine the luciferase activity.

### Statistical analysis

Statistical Product and Service Solutions (SPSS) 20.0 software (IBM, Armonk, NY, USA) was used for statistical analysis. All experiments were repeated 3 times, and the quantitative results were expressed by mean ± SD (X ± S). The quantitative values between the two groups were compared using independent sample t-test, the quantitative values between multiple groups were compared using one-way ANOVA, and the comparison between the two groups was conducted using S-N-K method. P<0.05 was considered statistically significant.

## Results

### Expression of miR-9-5p and TnC in OA model

**Figure 1:**
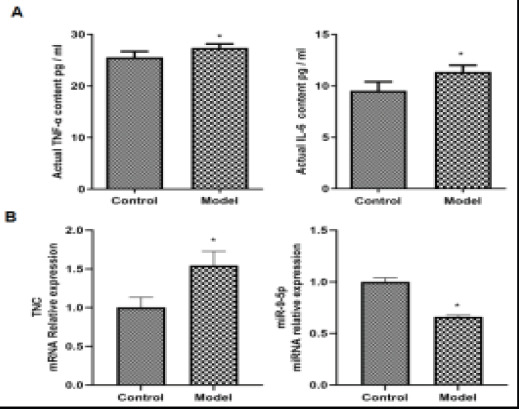
(A) The TNF-α and IL-6 expressions were detected with ELISA. (B) The relative mRNA levels of miR-9-5p and TnC were detected by quantitative real-time PCR. The results were shown as the mean ± SEM, 3 independent experiments, *P<0.05, compared with the control

To study the possible significance of miR-9-5p in OA, select IL-1β Stimulate chondrocytes, the expression levels of TNF-α and IL-6 were determined by Enzyme-Linked Immunosorbent Assay. And beyond that, the expression levels of miR-9-5p and TnC were detected by quantitative Real-time PCR. Compared with control, the expression levels of TNF-α and IL-6 were upregulated in IL-1β incubated chondrocytes (Figure 1A). Compared with control, the expression levels of TnC was upregulated, meanwhile, the expression levels of miR-9-5p was downregulated (Figure 1B).

### Cell transfection

**Figure 2:**
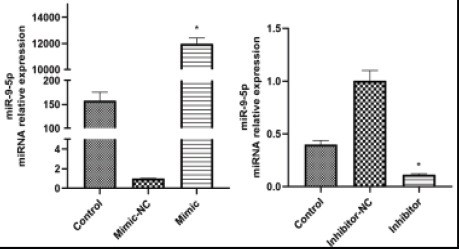
The relative mRNA levels of miR-9-5p were detected by quantitative real-time PCR. The results were shown as the mean ± SEM, 3 independent experiments, *P<0.05, compared with NC

To verify the transfection effect, the expression levels of miR-9-5p were detected by quantitative Real-time PCR. Compared with mimic-NC, the expression levels of miR-9-5p was increased significantly in mimic+Model. In inhibitor+Model, however, compared with inhibitor-NC, the expression levels of miR-9-5p was decreased significantly (Figure 2).

### MiR-9-3p inhibits IL-1β induced chondrocyte apoptosis

**Figure 3:**
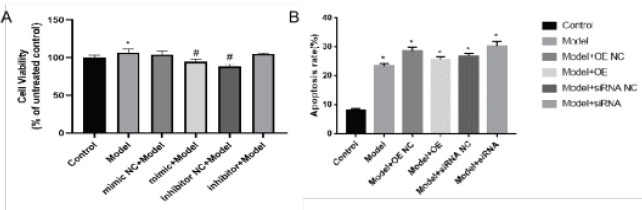
Suppression of miR-9-5p inhibited IL-1β-induced apoptosis. (A) Determination of chondrocyte viability by CCK-8 analysis; (B) Determination of chondrocyte apoptosis by flow cytometry. The results were shown as the mean ± SEM, 3 independent experiments, *P<0.05, compared with control, #P<0.05, compared with Model

To further clarify the function of miR-9-5p in OA, we transfect miR-9-5p mimic NC, miR-9-5p mimic, miR-9-5p inhibitor NC and miR-9-5p inhibitor into IL-1β in treated chondrocytes (Figure 3A, 3B). The results of CCK-8 and flow cytometry indicated that inhibitor of miR-9-5p significant inhibition of IL-1β Induced chondrocyte apoptosis.

### MiR-9-5p targets and regulates TnC expression

**Figure 4:**
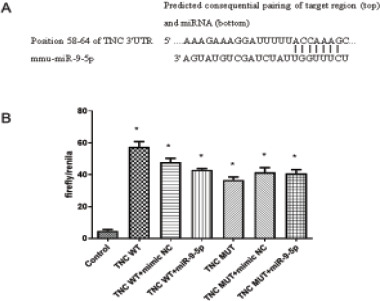
TnC targeted miR-9-5p in chondrocyte. (A) The possible binding sites between TnC and miR-9-5p from Starbase; (B) The luciferase activities was examined using luciferase reporter assay. *P<0.05, compared with control

In our previous study we found that overexpression of miR-9-5p in OA mice can down regulate TnC to inhibit chondrocyte apoptosis and promote cartilage remodeling16. Online prediction identified the targeted binding site between miR-9-5P and the 3′-UTR of TnC mRNA, we found that TnC contains potential complementarities of miR-9-3p (Figure 4A). Therefore, we want to further understand the relationship between miR-9-5p and TnC. Dual luciferase reporter gene assays revealed that miR-9-3p mimic reduced the luciferase activity of TnC WT group, however, no difference in TnC MUT group (Figure 4B). In general, these results confirm that miR-9-5p targets and regulates TnC expression into IL-1β in treated chondrocytes.

### MiR-9-5p can inhibits chondrocyte apoptosis by negatively targeting TnC

**Figure 5:**
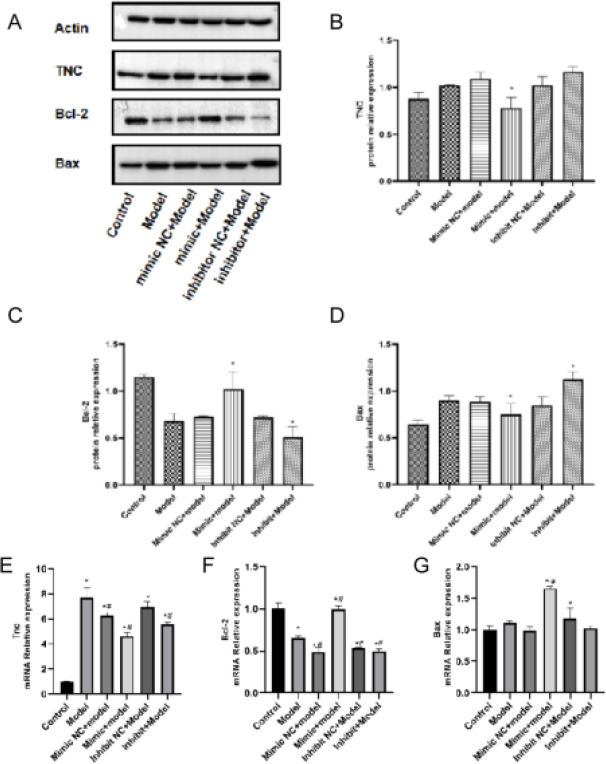
MiR-9-5p can inhibits chondrocyte apoptosis by negatively targeting TnC. MiR-9-5p mimic NC, miR-9-5p mimic, miR-9-5p inhibitor NC and miR-9-5p inhibitor transfected into IL-1β treated chondrocytes, (A) The expression of protein ((B) TnC, apoptosis related genes (C) Bcl-2 and (D) Bax) were detected by WB. The relative mRNA levels ((E) TnC, apoptosis related genes (F) Bcl-2 and (G) Bax) were detected by qRT-PCR. The results were shown as the mean ± SEM, 3 independent experiments, *P<0.05, compared with control, #P<0.05, compared with Model

To clarify the relationship between miR-9-5p and TnC, we transfected miR-9-5p mimic NC, miR-9-5p mimic, miR-9-5p inhibitor NC and miR-9-5p inhibitor into IL-1β treated chondrocytes, TnC and apoptosis related genes (Bcl-2, Bax) were detected by quantitative Real-time PCR and WB (Figure 5A). The results of qRT-PCR and western blot indicated that the mRNA and protein level of TnC were decreased significantly in mimic+Model as compared with Model (Figure 5B, 5E), the mRNA and protein level of Bcl-2 were increased significantly in mimic+Model as compared with Model (Figure 5C, 5F), the protein level of Bax was decreased significantly in mimic+Model as compared with Model (Figure 5D); The protein level of TnC was increased in inhibitor+Model as compared with Model (Figure 5B), the mRNA and protein level of Bcl-2 were decreased significantly in inhibitor+-Model as compared with Model (Figure 5C, 5F), the protein level of Bax was increased significantly in inhibitor+Model as compared with Model (Figure 5D). In summary, miR-9-5p can inhibits cell apoptosis by negatively targeting TnC.

### Upregulation of miR-9-5p in OA can reduce the expression of inflammatory factors

**Figure 6:**
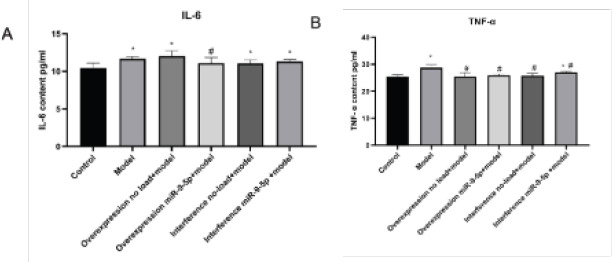
MiR-9-5p can reduce the expression of inflammatory factors in OA. Overexpression no-load, overexpression miR-9-5p, interference no-load and interference miR-9-5p transfected into IL-1β treated chondrocytes, The expression levels of inflammatory factors (A) TNF-α and (B) IL-6 were determined by ELISA

To clarify the functions of miR-9-5p in OA, we transfected overexpression no-load, overexpression miR-9-5p, interference no-load and interference miR-9-5p into IL-1β in treated chondrocytes. The expression levels of TNF-α and IL-6 were determined by ELISA. The results of ELISA indicated that the expression of TNF-α and IL-6 were decreased significantly in overexpression miR-9-5p group (Figure 6A, 6B). Thus, upregulation of miR-9-5p decreasing the expression of inflammatory factors (TNF-α, IL-6).

## Discussion

Research has proved that chondrocyte apoptosis is the key to the development of OA^17^. The relationship between apoptosis and pathogenesis of OA is still the focus of future research. It is reported that MicroRNA is an important participant in OA^18^. Mirnas may regulate genes, so mirnas may be potential targets for the treatment of OA. A large number of miRNAs have recently been found in OA joint tissue, such as miR-138-5p, miR-146a-5p, miR-335-5p and miR-9-5p, with miR-9-5p as the most valuable one^15^. The increase of miR-9 inhibits cell proliferation and survival of chondrocyte progenitors and articular chondrocytes, miR-9-5p is increased in the diseased cartilage of OA^19^. Upregulation of miR-9-5p may be the cause of chondrocyte apoptosis, which is the typical sign of OA. TnC is an extracellular matrix protein, which is a hexasaccharide protein component of ECM^20^. The expression of TnC is higher in the cartilage and perichondrium during fetal embryonic development, while lower in the adult cartilage and perichondrium^21^,^22^.

However, the expression of TnC is increased after tissue injury^23^. The literature shows that the expression of TnC is increased in the diseased cartilage and synovium of OA^24^, but the expression of TnC will be significantly reduced after the recovery of the damaged tissue^25^, so it indicates that TnC is involved in cartilage remodeling. Our previous research shows that the overexpression of miR-9-5p inhibits the apoptosis of chondrocytes by inhibiting the expression of TnC in the OA mice model^16^. Our research shows that miR-9-5p targets TnC expression and regulates OA progress.

## Limitation and innovation

Based on previous research, it has been proved in many dimensions such as tissue, cell, animal and animal informatics that miR-9-5p regulates target gene Tnc, which plays an important role in the development of osteoarthritis after tibial plateau fracture by inhibiting chondrocytes apoptosis. As with the majority of studies, the design of the current study is subject to limitations. The upstream regulatory mechanism that causes abnormal expression of miR-9-5p in disease groups and the downstream regulatory mechanism of Tnc inhibiting apoptosis in soft tissue cells are still unknown. All limit the clinical conversion of research results.

## Conclusions

In conclusion, these results suggested that miR-9-5p plays an important role in OA development, miR-9-5p can inhibits cell apoptosis by negatively targeting TnC. Thus, our findings demonstrated that the role of miR-9-5p in OA and helped clarify the potential use of biomarkers and potential therapeutic targets.

**Limitation and innovation:** Based on previous research, it has been proved in many dimensions such as tissue, cell, animal and animal informatics that miR-9-5p regulates target gene Tnc, which plays an important role in the development of osteoarthritis after tibial plateau fracture by inhibiting chondrocytes apoptosis. As with the majority of studies, the design of the current study is subject to limitations. The upstream regulatory mechanism that causes abnormal expression of miR-9-5p in disease groups and the downstream regulatory mechanism of Tnc inhibiting apoptosis in soft tissue cells are still unknown. All limit the clinical conversion of research results.
